# Supplementary figures and images for: The interaction of child abuse and rs1360780 of the FKBP5 gene is associated with amygdala resting‐state functional connectivity in young adults
Source: Hum Brain Mapp. 2021 Apr 5;42(10):3269–81. doi: 10.1002/hbm.25433 (PMC8193540; doi:10.1002/hbm.25433)

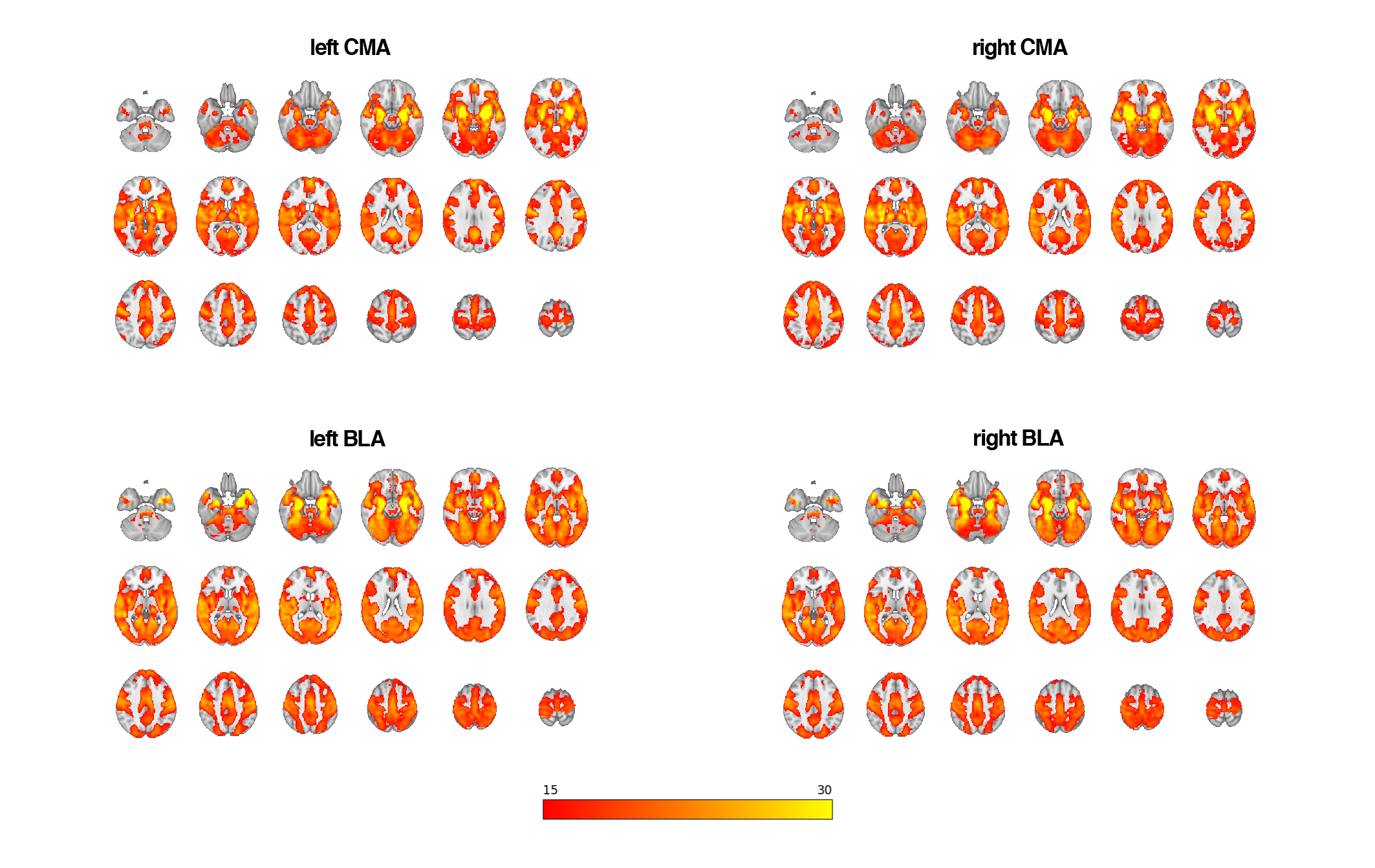

Supplement: Supplementary file 1 — Supplemental Figure 1 Average seed‐based functional connectivity for the centromedial and basolateral amygdala across all participants (uncorrected, but arbitrarily thresholded at t > 15). Overlaid on the MNI standard brain. Brains are displayed in radiological convention (i.e., left hemisphere is on the right side of the image and vice versa). [file HBM-42-3269-s001.tif]
